# Preprocedural Prediction Model for Contrast‐Induced Nephropathy Patients

**DOI:** 10.1161/JAHA.116.004498

**Published:** 2017-02-03

**Authors:** Wen‐jun Yin, Yi‐hu Yi, Xiao‐feng Guan, Ling‐yun Zhou, Jiang‐lin Wang, Dai‐yang Li, Xiao‐cong Zuo

**Affiliations:** ^1^ Clinical Pharmacy and Pharmacology Research Institute The Third Xiangya Hospital of Central South University Changsha Hunan China; ^2^ Xiangya School of Medical Science of Central South University Changsha Hunan China

**Keywords:** contrast media, contrast‐induced nephropathy, percutaneous coronary intervention, risk factor, risk prediction, Clinical Studies, Percutaneous Coronary Intervention, Computerized Tomography (CT), Epidemiology, Risk Factors

## Abstract

**Background:**

Several models have been developed for prediction of contrast‐induced nephropathy (CIN); however, they only contain patients receiving intra‐arterial contrast media for coronary angiographic procedures, which represent a small proportion of all contrast procedures. In addition, most of them evaluate radiological interventional procedure‐related variables. So it is necessary for us to develop a model for prediction of CIN before radiological procedures among patients administered contrast media.

**Methods and Results:**

A total of 8800 patients undergoing contrast administration were randomly assigned in a 4:1 ratio to development and validation data sets. CIN was defined as an increase of 25% and/or 0.5 mg/dL in serum creatinine within 72 hours above the baseline value. Preprocedural clinical variables were used to develop the prediction model from the training data set by the machine learning method of random forest, and 5‐fold cross‐validation was used to evaluate the prediction accuracies of the model. Finally we tested this model in the validation data set. The incidence of CIN was 13.38%. We built a prediction model with 13 preprocedural variables selected from 83 variables. The model obtained an area under the receiver‐operating characteristic (ROC) curve (AUC) of 0.907 and gave prediction accuracy of 80.8%, sensitivity of 82.7%, specificity of 78.8%, and Matthews correlation coefficient of 61.5%. For the first time, 3 new factors are included in the model: the decreased sodium concentration, the INR value, and the preprocedural glucose level.

**Conclusions:**

The newly established model shows excellent predictive ability of CIN development and thereby provides preventative measures for CIN.

## Introduction

Contrast‐induced nephropathy (CIN) is an important cause of acute kidney injury (AKI) in both ambulatory and hospitalized patients. With the wide use of contrast media (CM), CIN has become the third prevalent cause of all hospital‐acquired renal failure, accounting for 10%.^1^ Furthermore, the development of CIN has been reported to prolong hospitalization and increase mortality and morbidity.^2^ The precise pathophysiological mechanism of CIN remains unclear, but some studies have shown the pathogenesis of CIN to be related to the toxicity effect of CM on the tubular epithelial cells due to apoptosis, disturbances in intrarenal hemodynamics, and medullary hypoxia.^3^

Unfortunately, few strategies have been shown to prevent and cure CIN effectively. Therefore, it is important to comprehensively assess the risks of CIN before CM administration and to take preventative measures. Preexisting chronic kidney disease and diabetes mellitus are the most important risk factors for CIN. Age over 70, preprocedural dehydration, congestive heart failure, anemia, volume and type of CM administered, and concurrent administration of nephrotoxic drugs were found to be potential risk factors.^4^, ^5^ A number of risk prediction models with many important predisposing factors have been developed for the evaluation of an individual patient's risk of developing CIN. However, these models have exclusively focused on populations receiving intra‐arterial CM for coronary angiographic procedures, and no model developed a predictive approach for more common contrast‐enhanced computed tomography (CT) procedures.^6^ Indeed, the risk of CIN in a low‐risk population given intravenous contrast‐enhanced CT procedures is not small.^7^ What is more, most of the models evaluate the radiological interventional procedure‐related variables; thus, they complete risk assessment only after CM administration. As far as we know, there have been only 4 published models that studied the risk factors before coronary angiography.^8^, ^9^, ^10^, ^11^ Among the 4 models, only Liu et al^11^ developed a preprocedural model in 728 Chinese patients with chronic total occlusion undergoing percutaneous coronary intervention, but common variables such as those related to diabetes were not included in the model, so it is not particularly applicable to a diabetes population. In addition, all of the 4 models are focused on coronary angiography, and thus, they are incapable of predicting CIN before other CM procedures such as intravenous contrast‐enhanced CT.

The purpose of this study was to determine the incidence and to assess predictive factors of CIN in Chinese patients and to develop a predictive model that could provide a good prediction for CIN before patients were exposed to CM.

## Materials and Methods

### Ethics Statement

The study protocol was approved by the Medical Ethical Committee in the Third Xiangya Hospital of Central South University (No. 2016‐S160). All subjects were anonymized so informed consent was not required. This study conformed to the ethical guidelines of the 1975 Declaration of Helsinki and Strengthening the Reporting of Observational Studies in Epidemiology (STROBE) guidelines.

### Patient Population

We performed a retrospective single‐center case control study in hospitalized adults from September 2007 to January 2015. According to our institute protocol, patients were included if they were treated with CM for coronary angiography or percutaneous coronary intervention or received intravenous CM such as for CT or endovascular procedures (n=69 827), identified by the electronic medical record system at the Third Xiangya Hospital of Central South University, Changsha, China. The exclusion criteria were preprocedure estimated glomerular filtration rate (eGFR) under 15 mL/(min·1.73 m^2^) (n=422), age ≤15 (n=635), missing variables more than 30%, which means that the number of missing variables is greater than 25 in the 83 variables (n=4914), and without serum creatinine value within 14 days before CM procedures or within 72 hours after procedures (n=55 478).

Their detailed demographic and clinical characteristics were collected from the hospital information system. The serum creatinine (Scr) concentration at the earliest within 14 days before a procedure was defined as the baseline, and the highest Scr within 72 hours after the procedure was used as the follow‐up Scr to evaluate the incidence of CIN.

### Definition

In this study, CIN was defined as an increase of Scr of 0.5 mg/dl (44.2 μmol/L) or 25% relative increase in serum creatinine from the baseline value to 72 hours after exposure to CM in the absence of alternative causes for acute kidney injury according to the Contrast Media Safety Committee (CMSC). Creatinine clearance was calculated using the modification of diet in renal disease (MDRD) equation, and chronic kidney disease was defined as eGFR<60 mL/(min·1.73 m^2^) as estimated with the modified MDRD formula.^12^, ^13^ Anemia is defined as hemoglobin (HGB) concentration <13 g/dL for men and <12 g/dL for women.^14^

### Statistical Analysis

Continuous variables of each group are presented as mean±standard deviation, and the categorical variables are expressed as absolute values and percentages. A t test was used to compare the normally distributed continuous variables; otherwise, the Mann‐Whiney U test was used. Categorical variables were performed by chi‐squared test. A 2‐tailed value of *P*<0.05 was established as the threshold of statistical significance. Data analysis was performed with the statistical package SPSS, version17.0 (SPSS Inc, Chicago, IL).

### Prediction Model Development

We developed the prediction model based on machine learning. Randomization and data analysis were performed using random forests (RF), an ensemble of decision trees.^15^ RF is good at describing the relationship between independent and dependent variables with high flexibility and sufficient accuracy. The 2 main parameters in RF are mtry, the number of input variables randomly chosen at each split, and ntree, the number of trees in the forest. In this model, the mtry is 4 and the ntree is 1000. The training group is used to form the algorithm composed of 1000 trees, each of which is constructed using the bootstrap samples from the training data and random feature selection. Each node is best split from a random selected set. When the RF algorithm best separates all instances and this tree is able to classify all instances, this node becomes a terminal node with each unpruned tree grown to its maximum extent. After 1000 trees are achieved, the majority vote of all analogous trees in the forest was taken for the predictions for test data. RF was implemented by the RF function in the R package (ver 4.6.7).

The selected 8800 patients were randomly divided into 2 separate data sets: 80% of the patients (CIN=942, non‐CIN=6098) in our database were selected to the training data set (the algorithm creation group), and the remaining 20% (CIN=231, non‐CIN=1529) were reserved as the external validation sets (validation group) to obtain unbiased estimates of correct classification rates and variable importance.

In this model RF is also used to assess the importance of variables in model quality when each variable is replaced in turn by random noise in each tree. The variable importance is measured by the resulting deterioration in model quality. The deterioration in model quality can be assessed by the change in misclassification rates for the out‐of‐bag validation. The heuristics was based on the Gini criterion. Specifically, we recorded the decrease in the Gini node impurity for the variable Xj, which was used to form the split at each split. In the forest where Xj formed the split, the average of all decreases in the Gini impurity decided the Gini variable's importance. In addition, the AUC was also used to assess the importance of selected variables: when a variable is excluded in the model, the larger the change in value of AUC is, the more important the variable is.

Five‐fold cross‐validation was primarily used as an internal validation to evaluate the prediction accuracies of the model. Briefly, we split the data set into 5 roughly equal‐sized parts, and then 4 of them were fit into the model while the other part was used to calculate the error rate. The process was repeated 5 times so that every part could be predicted as a validation set.

The prediction performance was assessed by several criteria including the overall prediction accuracy (R), sensitivity (SE), specificity (SP), and Matthews correlation coefficient (MCC). The equations are as follows:

$$\text{SE}=\frac{\text{TP}}{\text{TP}+\text{FN}}$$

$$\text{SP}=\frac{\text{TN}}{\text{TN}+\text{FP}}$$

$$R=\frac{\text{TP}+\text{TN}}{\text{TP}+\text{FP}+\text{TN}+\text{FN}}$$

$$\text{MCC}=\frac{\text{TP}\times \text{TN}-\text{FN}\times \text{FP}}{\sqrt{(\text{TP}+\text{FN})(\text{TP}+\text{FP})(\text{TN}+\text{FN})(\text{TN}+\text{FP})}}$$

where true positive (TP) is the number of positive samples predicted correctly, true negative (TN) is the number of negative samples predicted correctly, false positive (FP) is the number of negative samples indicated as positive, and false negative (FN) is the number of positive samples indicated as negative.

SE and SP allow computation of the percentage of correctly predicted CIN and non‐CIN, respectively, while prediction accuracy means percentage of correctly predicted CIN and non‐CIN. MCC is the statistical parameter to assess the quality of prediction and to take care of the data unbalancing. The Matthews correlation coefficient ranges from −1 to 1. MCC=1 indicates the best possible prediction, and MCC=−1 points out the worst possible prediction.

## Results

### Patient Characteristics

Of a total of 69 827 patients administrated CM, 8800 patients (5468 men, 3332 women; mean age 55.3±14.8 years) were included in this study. Of them, 1173 (13.3%) developed CIN. Table 1 shows the clinical characteristics of patients who developed CIN and of those who did not show this complication after CM administration.

**Table 1:** Demographic, Clinical, and Angiographic Data in CIN and Non‐CIN Patients of the Training Data Set

Variable	Non‐CIN (n=6098)	CIN (n=942)	*P* Value
Sex (male)	3801 (62.33%)	609 (64.65%)	0.171
Age (y)	55.23±14.81	55.87±15.57	0.215
eGFR (mL/[min·1.73 m^2^])	103.03±49.07	116.82±74.97	0.000*
CKD	791 (12.97%)	170 (18.05%)	0.000*
Diabetes	816 (13.38%)	68 (7.22%)	0.000*
Mechanical ventilation	808 (13.25%)	148 (15.71%)	0.040*
Myocardial infarction	330 (5.41%)	42 (4.46%)	0.224
Shock	147 (2.41%)	44 (4.67%)	0.000*
Gout	66 (1.08%)	5 (0.53%)	0.115
Liver cirrhosis	322 (5.28%)	103 (10.93%)	0.000*
Kidney transplant	42 (0.69%)	1 (0.11%)	0.033*
Atherosclerosis	796 (13.1%)	99 (10.51%)	0.029*
ICU admission before procedure	568 (9.31%)	145 (12.85%)	0.000*
Coronary heart disease	371 (6.08%)	43 (4.56%)	0.065
Anemia	3858 (63.27%)	667 (70.81%)	0.000*
Systolic blood pressure	126.97±27.77	128.70±21.32	0.704
Diastolic blood pressure	78.36±12.65	77.00±12.05	0.606
Blood glucose	6.47±2.92	7.07±4.36	0.000*
RDW	46.05±7.23	46.98±7.82	0.000*
NEUT%	75.15±12.85	76.59±12.92	0.000*
NEUT	7.80±5.36	8.26±5.79	0.033*
Lymphocytes%	16.35±10.33	15.13±10.19	0.000*
Lymphocytes	1.30±0.93	1.25±1.08	0.000*
Monocytes%	6.37±3.12	6.15±3.24	0.000*
Monocytes	0.58±0.48	0.57±0.38	0.240
Platelets	198.77±112.32	184.44±112.83	0.000*
Mean corpuscular volume	91.25±7.60	91.51±7.27	0.440
Mean corpuscular hemoglobin	29.76±3.06	29.84±2.90	0.740
Mean corpuscular hemoglobin concentration	326.06±19.44	325.83±18.90	0.781
Mean platelet volume	11.05±1.47	11.11±1.43	0.107
Eosinophils%	1.54±2.16	1.41±2.02	0.054
Eosinophils	0.12±0.21	0.11±0.17	0.033*
Basophil%	0.32±0.41	0.30±0.41	0.009*
Basophil	0.02±0.04	0.03±0.05	0.329
Reticolociti%	1.59±0.76	1.59±0.70	0.000*
Reticolociti	3.80±6.16	5.14±8.71	0.000*
Platelet distribution width	14.92±2.67	14.95±2.61	0.074
Platelet cell ratio	0.23±0.11	0.22±0.12	0.000*
Total bilirubin	32.65±59.44	37.15±74.15	0.808
Albumin	35.12±7.25	33.64±7.26	0.000*
Macro‐platelet cell ratio	36.69±7.97	36.95±7.69	0.355
Albumin: globulin ratio	1.34±0.35	1.31±0.37	0.023*
Creatinine	88.26±86.04	122.93±137.48	0.000*
Total protein	62.06±10.91	60.19±11.93	0.011*
Globulin	27.38±6.26	27.02±6.92	0.100
Alanine aminotransferase	67.44±161.33	72.64±150.05	0.353
Urea	6.16±4.66	8.23±7.56	0.000*
Uric acid	258.82±130.17	271.21±146.68	0.009*
Direct bilirubin	19.03±44.45	22.57±54.00	0.715
Total bile acids	15.31±38.35	18.44±43.96	0.133
Aspartate amino transferase	71.46±191.95	94.95±201.63	0.027*
Chlorine	102.97±5.36	102.97±5.88	0.984
Sodium	137.91±5.60	135.88±9.97	0.000*
Potassium	4.08±0.52	4.16±0.60	0.000*
Calcium	2.77±5.61	3.08±13.85	0.232
Hemoglobin	114.43±26.36	108.60±27.62	0.000*
Hematocrit	35.02±7.67	33.25±8.27	0.000*
Total cholesterol	4.23±1.52	4.18±1.78	0.238
LDL	2.33±1.06	2.23±1.13	0.008*
HDL	1.07±0.46	0.99±0.45	0.000*
Triglycerides	1.75±2.35	2.24±4.23	0.000*
Thrombin time	16.16±11.03	17.12±15.02	0.018*
Prothrombin time	12.89±4.01	13.51±4.86	0.000*
Fibrinogen	3.67±1.25	3.60±1.29	0.304
APTT	47.88±38.01	33.074±13.29	0.409*
International normalized ratio	1.11±0.35	1.17±0.503	0.000*
Pulse	82.49±14.43	85.72±17.13	0.000*
Diuretic	1760 (28.86%)	317 (33.65%)	0.003*
ACEI	695 (11.38%)	86 (9.13%)	0.039*
ARB	248 (4.07%)	26 (2.76%)	0.054*
NSAIDs	555 (9.10%)	76 (8.07%)	0.301
Vitamin C	2381 (39.04%)	363 (38.53%)	0.765
Alprostadil	690 (11.32%)	125 (13.27%)	0.081
Dopamine	268 (4.39%)	54 (5.73%)	0.067*
Cephalosporin	1173 (19.24%)	201 (21.34%)	0.000*
Glycopeptides	162 (2.66%)	30 (3.18%)	0.354
Quinolone	373 (6.12%)	65 (6.90%)	0.354
Vancomycin	49 (0.80%)	4 (0.42%)	0.211
Acyclovir	47 (0.77%)	2 (0.21%)	0.550
Aminoglycoside	643 (10.54%)	96 (10.19%)	0.742
Statins	882 (14.46%)	98 (10.40%)	0.001*
Asipirin	851 (13.96%)	100 (10.62%)	0.005*

ACEI indicates angiotensin‐converting enzyme inhibitor; APTT, activated partial thromboplastin time; ARB, angiotensin receptor blocker; CIN, contrast‐induced nephropathy; CKD, chronic kidney disease (defined as eGFR <60 mL/[min·1.73 m^2^); eGFR, estimated glomerular filtration rate; HDL, high‐density lipoprotein cholesterol; ICU, intensive care unit; LDL, low‐density lipoprotein cholesterol; NSAIDs, nonsteroidal anti‐inflammatory drugs; PT, prothrombin time; RDW, red blood cell distribution width; TG, triglyceride; TT, thrombin time.

*P<0.05.

**Figure 1:**
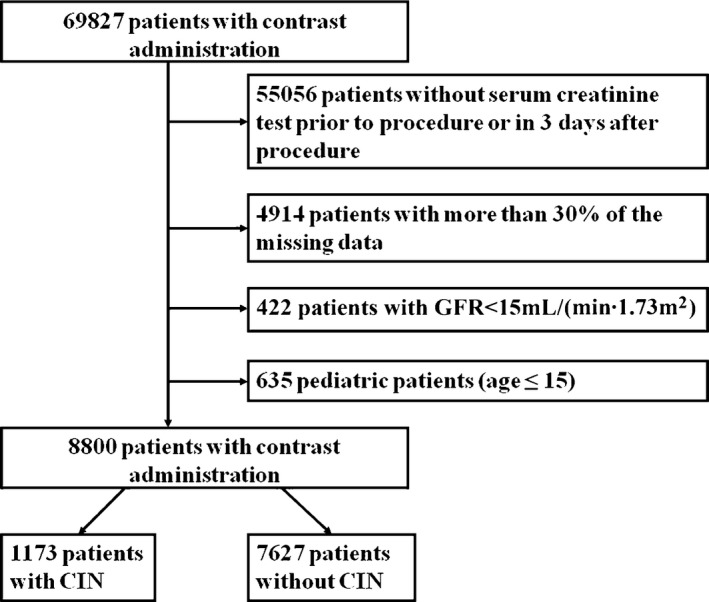
Flow chart depicting number of patients who were included in analysis after exclusion criteria. The total included encounters were divided into those with and without contrast‐induced nephrotoxicity (CIN). GFR indicates glomerular filtration rate.

Eighty‐three variables including demographic information, comorbidities, medications, and laboratory values were collected for each patient. Among the 8800 patients, there were 1656 patients undergoing percutaneous coronary intervention, and of them, 195 (11.8%) suffered from CIN. The incidence in the other CM procedures can be seen in Table 2. Figure 1 shows the number of patients included in analysis after exclusion criteria had been applied. The baseline characteristics of the training cohorts (7040 patients) separated by CIN and non‐CIN status are presented in Table 1. The mean baseline eGFR in 7040 patients was 104.9 mL/(min·1.73 m^2^) (SD 53 mL/[min·1.73 m^2^]). Chronic kidney disease was present in 962 patients (13.7%).

**Table 2:** The Incidence of CIN in Different CM Procedures

	CIN	Non‐CIN	Incidence
Intravenous contrast‐enhanced CT	734	4600	13.8%
Percutaneous coronary intervention	195	1464	11.8%
CT angiography	77	408	15.9%
Noncoronary angiography	32	176	15.4%
Other CM procedures	135	979	12.1%
Total	1173	7627	13.3%

CIN indicates contrast‐induced nephropathy; CM, contrast media; CT, computed tomography.

### Variables of Importance

**Figure 2:**
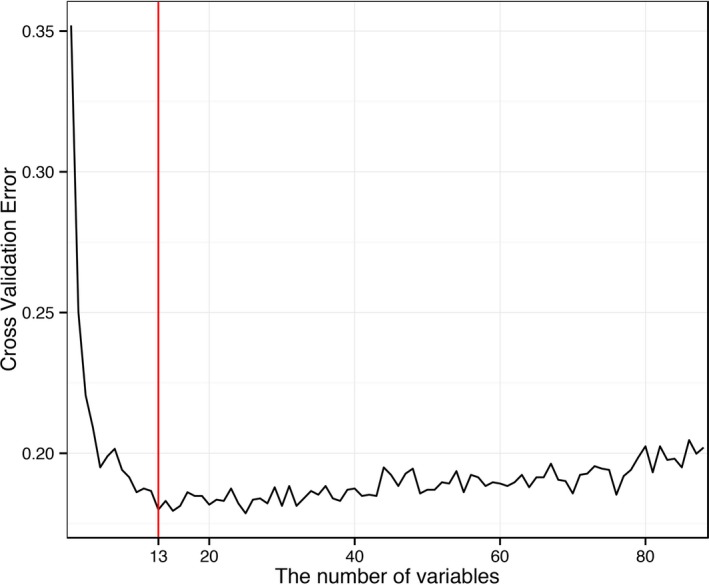
The relationship between the cross‐validation error and the number of variables.

In general, as more variables are chosen, the error of the model will be smaller. However, increasing the number of variables does not benefit clinical practice. To identify the prominent features, we carried out variable selection using different feature subsets by the RF method. Figure 2 shows the relationship between the cross‐validation error and the number of variables. When the variables increase to 13, the error has a sharp decrease to 0.18. With the variables increasing gradually to 83, the error still remains at a similar level.

**Figure 3:**
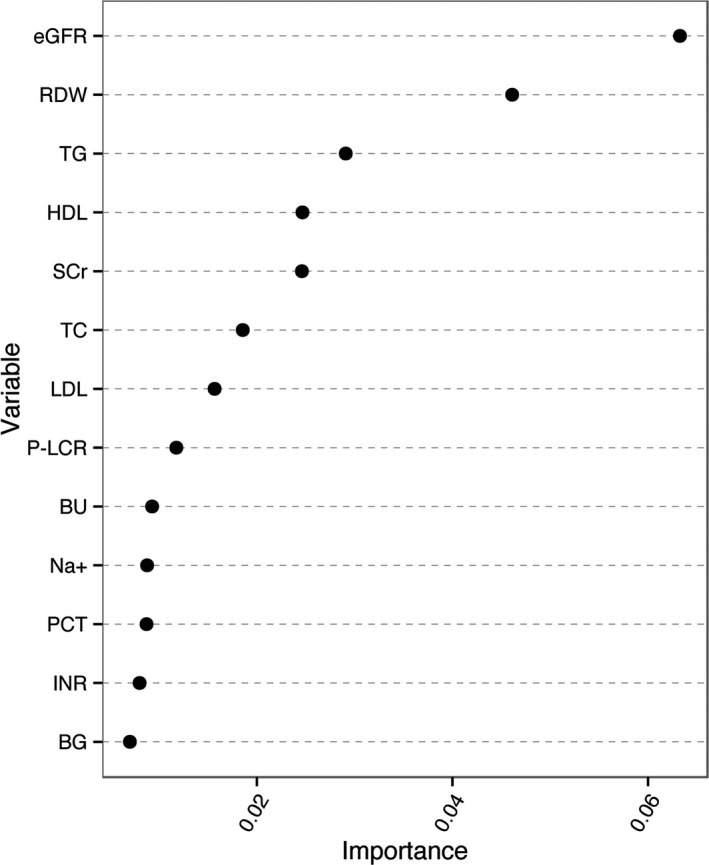
The importance of the 13 variables of the contrast‐induced nephropathy prediction model. BG indicates blood glucose; BU, blood urea; eGFR, estimated glomerular filtration rate; HDL, high density lipoprotein cholesterol; INR, International Normalized Ratio; LDL, low density lipoprotein cholesterol; Na+, serum sodium; PCT, plateletocrit; P‐LCR, platelet larger cell ratio; RDW, red cell distribution width; SCr, serum creatinine; TC, total cholesterol; TG, triglyceride.

Thus, our final model included 13 indispensable features for CIN prediction: baseline eGFR, red cell distribution width (RDW), triglycerides, the most recent serum creatinine before the procedure, high‐density lipoprotein cholesterol (HDL), total cholesterol, low‐density lipoprotein cholesterol (LDL), blood urea (BU), platelet larger cell ratio (P‐LCR), serum sodium (Na^+^), plateletocrit (PCT), international normalized ratio (INR), and blood glucose (BG). The importance of the 13 variables is demonstrated in Figure 3. The larger the importance number is, the more important the variable is. In addition, as shown in Table 3, the change of AUC value is also used to assess the importance of the 13 selected variables. There are some differences about the sorting on the importance of the variables as shown in Figure 3 and Table 3. In the first approach the serum creatinine is more important than serum sodium as shown in Figure 3. However, in the method based on AUC, the AUC change value of serum creatinine is bigger than that of serum sodium. Actually, the approach of the out‐of‐bag validation and Gini in Figure 3 pays more attention to the importance of the whole variables to the model, but the method of comparing the AUC change values after excluding each variable in Table 3 emphasizes the impact of individual variables on the model. Those differences do not affect the classification results.

**Table 3:** The Change of AUC Value When Each Variable Is Excluded in the Model

Variables	The Value of AUC After Excluding a Variable	The Change Value of AUC After Excluding a Variable
All	0.907	0.000
Baseline eGFR	0.860	0.047
Serum sodium	0.867	0.040
Red cell distribution width	0.870	0.037
Triglyceride	0.870	0.037
High‐density lipoprotein cholesterol	0.871	0.036
Low‐density lipoprotein cholesterol	0.873	0.034
Blood urea	0.873	0.034
Platelet–larger cell ratio	0.873	0.034
Blood glucose	0.874	0.033
Total cholesterol	0.876	0.031
International normalized ratio	0.876	0.031
Serum creatinine	0.877	0.030
Plateletocrit	0.878	0.029

AUC indicates area under the receiver‐operating curves; eGFR, estimated glomerular filtration rate.

### Classification Results

**Figure 4:**
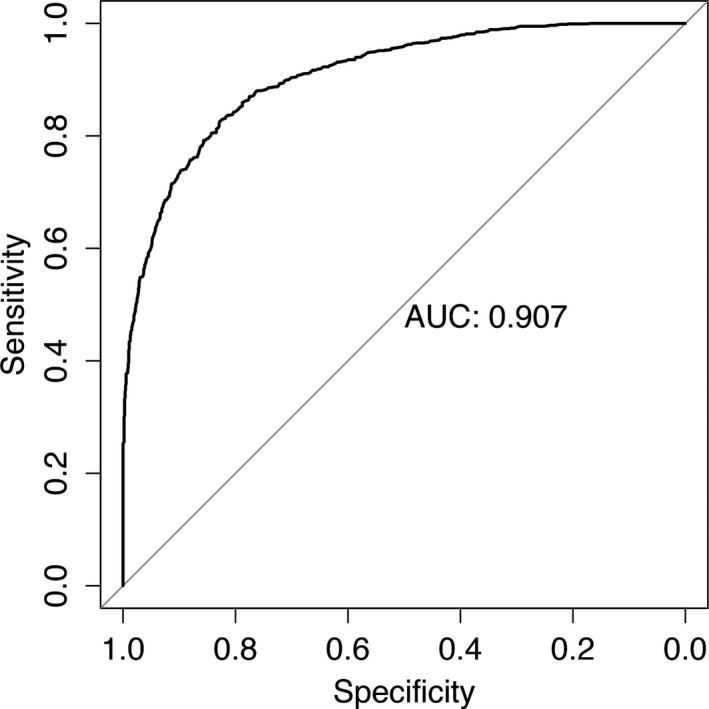
Cross‐validated receiver‐operating characteristic curves for predicting contrast‐induced nephropathy in the prediction model. AUC indicates area under the receiver‐operating curves.

Classification of a patient in the RF was determined by the number of votes from all classification trees in the forest. We obtained different sensitivity, specificity, and accuracy while changing the threshold of voting. The receiver‐operating curve (ROC) was developed on basis of the sensitivity and specificity of the above values. The area under the ROC curve (AUC) is often used as an additional performance index. The closer AUC was to 1, the greater was the predictive ability of the model. A model with no predictive ability would yield the diagonal line. Figure 4 shows the ROC curve for this model. It is significant that our model obtained an AUC of 0.907. Such results sufficiently indicated that a big separation for CIN and non‐CIN patients was indeed obtained from this prediction model.

The prediction accuracies of the model were internally evaluated by 5‐fold cross‐validation. On the average, the prediction model gave a prediction accuracy of 82.2%, sensitivity of 84.4%, specificity of 79.9%, and Matthews correlation coefficient of 64.4%.

In order to examine the performance of the newly developed model, we tested our training models based on a data set containing 231 patients with CIN and 1529 patients without CIN for an external validation. The basic information comparing the training and validation sets is showed in Table 4. The external validation achieved 82.4% for accuracy, 83.9% for sensitivity, 80.3% for specificity, and a Matthews correlation coefficient of 0.647, respectively. The result of high prediction accuracy and successful prediction suggested that the new model was efficiently used to predict CIN.

**Table 4:** Demographic and Clinical Data in Training and Validation Cohorts

Variable	Training Cohorts (n=7040)	Validation Cohorts (n=1760)	*P* Value
Sex (male)	4410 (62.64%)	995 (61.27%)	0.303
Age (y)	55.31±14.91	55.42±14.71	0.800
eGFR (mL/[min·1.73 m^2^])	104.86±53.47	105.19±60.73	0.222
CKD	961 (13.65%)	233 (14.35%)	0.463
Diabetes	884 (12.56%)	212 (13.05%)	0.587
RDW	46.17±7.32	46.08±7.31	0.138
Triglycerides	1.82±2.68	1.86±2.94	0.717
HDL	1.06±0.46	1.06±0.45	0.766
Creatinine	92.90±95.28	92.00±102.17	0.803
LDL	2.31±1.07	2.30±1.15	0.462
Platelet‐cell ratio	0.23±0.11	0.23±0.11	0.463
Urea	6.43±5.19	6.44±5.35	0.339
Sodium	137.64±6.40	137.71±6.26	0.811
Macroplatelet‐cell ratio	36.69±7.94	36.82±7.96	0.548
Coronary heart disease	414 (5.88%)	81 (4.99%)	0.162
International normalized ratio	1.12±0.38	1.12±0.47	0.736
Blood glucose	6.55±3.16	6.53±2.99	0.899

CKD indicates chronic kidney disease (defined as eGFR <60 mL/[min·1.73 m^2^); eGFR, estimated glomerular filtration rate; HDL, high‐density lipoprotein cholesterol; LDL, low‐density lipoprotein cholesterol; RDW, red blood cell distribution width.

## Discussion

With the increasing use of CM, CIN has become the third leading cause of hospital‐acquired acute renal failure, contributing to growing in‐hospital morbidity and mortality, hospitalization prolongation, and increase in costs.^16^, ^17^ Unfortunately, there are few definitively effective strategies for prophylaxis or treatment of CIN.^18^, ^19^ Therefore, it is necessary to establish a model involved in various comprehensive factors related to CIN that lets patients be protected from CM, especially for those who might be at high risk.

Several models have been developed for the prediction of CIN; however, they just focus on patients receiving intra‐arterial CM for coronary angiographic procedures, which represent only a small proportion of all contrast procedures.^8^, ^9^, ^10^, ^11^, ^20^, ^21^, ^22^, ^23^ In fact, contrast‐enhanced CT scans are much more commonly used, and the incidence of CIN resulting from contrast‐enhanced CT procedures is also high, occurring in 11% of an outpatient setting population.^7^ Thus, these prediction models might not be available for those who undergo these procedures, such as intravenous contrast‐enhanced CT and CT angiography. The Mehran risk score, a classic model for CIN, has widely been used for many years, but the risk factors included in the model occur only in patients receiving percutaneous coronary intervention. Furthermore, the volume of CM, a variable in this model, cannot be known before the procedure. So risk assessment cannot be completed before CM exposure. Although most of the previously established prediction models included both preprocedural and procedure‐related variables such as the volume of CM, few studies aimed to develop risk models for CIN before procedure. Liu et al^11^ developed a preprocedural model in a Chinese population with chronic total occlusion undergoing percutaneous coronary intervention including 3 periprocedural variables: age >75 years, LVEF <40%, and Scr >1.5 mg/dL. Some common risk factors are not included, such as diabetes, so it may not good at predicting CIN in the diabetes patients who are at higher risk of CIN. The other 3 models did not involve a Chinese population. Furthermore, all of these models also focus on coronary angiographic procedures, so they are only able to be used before coronary angiographic procedures.^8^, ^9^, ^10^, ^11^

Here, we developed a prediction model of CIN with preprocedure variables by RF, which was composed of Chinese patients administered CM. The new system was first established to provide a prediction model of contrast‐induced AKI using preprocedural variables in an unselected population. The AUC of this newly developed model was 0.907, demonstrating good discriminative power. Although the present model did not include procedure‐related variables, its predictive value was better than that of the Mehran risk score, whose AUC was 0.67. The prediction accuracies were internally evaluated by 5‐fold cross‐validation and tested by the test data set for an external validation. Thirteen of 83 variables were chosen in our risk prediction model for CIN. A strong relationship is found between decreased sodium and increased risk of CIN in patients who underwent CM administration. Additionally, INR is also observed to be a powerful factor affecting CIN prediction. In addition, we used the preprocedural glucose level in a CIN risk prediction model for the first time in the present study.

Our study is the first to show a relationship between decreased serum sodium and increased risk of CIN among patients who underwent CM administration. For 1 thing, hospital‐acquired lower serum sodium is found to coincide with various inflammatory conditions.^24^, ^25^ Inflammatory cytokines such as IL‐1β and IL‐6 have been reported as mediators in the development of hyponatremia related to ADH secretion.^25^, ^26^, ^27^ Inflammation is associated with impaired renal function. In addition, activation of the signaling pathway for inflammation by CM in human renal proximal tubular cells has been reported.^28^ Furthermore, some articles have shown that hyponatremia might be a surrogate marker for the severity of certain pathologies such as heart failure, pneumonia, and liver disease,^29^, ^30^ which may promote the development of CIN, so patients with lower plasma sodium are susceptible to CIN after CM exposure.

Our research indicates that baseline eGFR is an important risk factor, as has been found in previous studies.^16^, ^31^, ^32^ The eGFR of CIN patients was lower than that of non‐CIN patients in the previous studies because they got different eGFR values from using different preprocedural time points, such as the first admission time or 7 or 14 days before the procedure. However, in the present research, the eGFR from 14 days preprocedure of CIN is greater than that of non‐CIN patients. In addition, the most recent SCr before procedure is 1 of the strongest prediction factors for CIN development. The SCr of CIN is greater than that of non‐CIN patients, which is consistent with the previous studies. In addition, we also compared the increased value of Scr, defined as the most recent Scr value before CM procedures minus that at admission, between the CIN group and the non‐CIN group. This result showed that Scr increased 5.3% on average in the CIN group and decreased 5.2% in the non‐CIN group. In view of the above factors, the renal function of some patients is prone to be affected by medical interventions such as nephrotoxic agents and operations. Although these patients have a normal renal function at admission, Scr will increase rapidly under admission conditions and leave these patients more prone to develop CIN from CM.

Blood urea, a parameter for evaluation renal function, is also in our model, although the renal function is mainly evaluated by eGFR. Furthermore, blood urea plays a fundamental and direct role in fluid and sodium homeostasis regulated by neurohormonal systems.^33^, ^34^, ^35^, ^36^ The decreased intravascular effective volume and decompensated heart failure reduce the rate of urea excretion and increase blood urea levels.^37^, ^38^ The decreased intravascular effective volume would cause disturbances in intrarenal hemodynamics that potentially could result in CIN. Thus, blood urea levels provide an effective way to assess circulatory volume and play an important role in the prediction of CIN.

The RDW, reported routinely as part of an automated full blood count and used to evaluate the size of circulating red blood cells and the possible causes of anemia, is a main risk factor in the development of CIN. It has been found that RDW correlates with kidney function. Moreover, recent studies have reported an independent association between increased RDW and CIN in patients who underwent PCI.^39^, ^40^, ^41^ Mizuno et al added the RDW to the Mehran risk score for predicting CIN in patients with ST‐elevation acute myocardial infarction.^42^ Elevated RDW has been shown to be an effective biomarker for chronic inflammation and oxidative stress.^43^ Therefore, patients with an increased RDW may have a high level of oxidative stress and chronic inflammation, which may lead to renal dysfunction after CM administration.

Although several studies have indicated that elevated glucose level is a risk factor of CIN,^44^, ^45^, ^46^ our study found that the blood glucose level preprocedure enters the CIN risk prediction model in both diabetic and nondiabetic patients. Additionally, glycemic control using insulin in critically ill patients has been shown to reduce the rates of AKI.^47^, ^48^ The mechanism of the underlying relationship between acute hyperglycemia and the risk of CIN is still unknown. Studies demonstrate that elevated glucose levels are associated such factors as endothelial dysfunction,^49^ increased activation of prothrombotic factors,^50^, ^51^ markers of vascular inflammation,^52^, ^53^ and generation of reactive oxygen species.^54^, ^55^ An animal study has demonstrated that hyperglycemia exacerbates kidney damage through mitochondrial dysfunction.^51^ Such factors may lead to kidney impairment if patients are exposed to CM.

By examining the relationships among HDL cholesterol, LDL cholesterol, triglycerides, serum total cholesterol, and CIN, our study found that hypercholesterolemia, hypertriglyceridemia, or low HDL would raise the risk for CIN. Such blood lipid factors result in reducing the production of nitric oxide and increasing oxidative stress and inflammation in the kidney.^56^, ^57^, ^58^

For the first time, the elevated INR has been reported in a CIN prediction model. INR monitoring is essential during oral anticoagulation therapy to minimize bleeding complications and thrombotic events. INR elevation indicates that the glomerulus may hemorrhage, and red blood cell casts obstruct renal tubules.^59^ Thus, INR is an important risk factor for CIN.

We also find that platelet activity biomarkers may correlate with the development of CIN. Those reflecting the platelet reactivity, including platelet count (PC), platelet–larger cell ratio (P‐LCR), mean platelet volume (MPV), and platelet distribution width (PDW), were evaluated in this study. PC and P‐LCR, the index of the platelet reactivity, are significant variables in this model. Thrombocytopenia has often been cited as an indicator of critical illness severity,^60^, ^61^ and a novel association between thrombocytopenia and postoperative AKI has been established.^61^ Activated platelets have been found as a source of vasoactive inflammatory mediators related to the endothelial integrity,^62^ which is a key player in the development of CIN.

## Conclusion

A risk prediction model with excellent predictive ability for CIN in Chinese patients has been successfully established. This model can be applied to patients administered CM for coronary procedures and other contrast procedures such as intravenous contrast‐enhanced CT, CT angiography, and noncoronary angiography. For the first time, there are 3 new factors included in the model: the decreased sodium concentration, the INR value, and the preprocedural glucose level.

### Limitations

The potential limitations of our study should be mentioned. First, this study is limited by its retrospective design, whose inherent weakness cannot be avoided. Second, our prediction model is derived and validated by a single center. For the wide application of the prediction model, it still needs to be validated in a multicenter trial. Third, any variable that was missing for more than 30% of the population was not assessed in the present study. Finally, we ignored unstructured clinical notes. Future studies addressing these limitations are necessary.

## Author Contributions

Yin and Zuo conceived and designed the study. Yin, Zuo, and Yi performed data acquisition and statistical analyses. Yin and Guan managed the patient database. All authors were involved in the data interpretation and discussion of the results. Yin, Wang, and Li prepared the figures. Yin and Zuo drafted the manuscript. All authors approved the final version of the manuscript.

## Sources of Funding

This study was supported by the Program for New Century Excellent Talents in University (NCET‐13‐0605), the New Xiangya Talent Project of the Third Xiangya Hospital of Central South University (No. 20150218), National Natural Science Foundation of China (81102512), and the National Clinical Pharmacy Key Specialty Construction Project.

## Disclosures

None.
